# Thyroid Transcriptomics Revealed the Reproductive Regulation of miRNA in the Follicular and Luteal Phases in Small-Tail Han Sheep with Different FecB Genotypes

**DOI:** 10.3390/genes14112024

**Published:** 2023-10-30

**Authors:** Cheng Chang, Xiaoyun He, Ran Di, Xiangyu Wang, Miaoceng Han, Chen Liang, Mingxing Chu

**Affiliations:** 1 State Key Laboratory of Animal Biotech Breeding, Institute of Animal Science, Chinese Academy of Agricultural Sciences (CAAS), Beijing 100193, China; changcheng20200911@163.com (C.C.); hexiaoyun@caas.cn (X.H.); diran@caas.cn (R.D.); wangxiangyu@caas.cn (X.W.); 2 College of Animal Science, Shanxi Agricultural University, Jinzhong 030801, China; hanmiaoceng@163.com

**Keywords:** small-tail Han sheep, follicular phase, luteal phase, thyroid gland, litter size, miRNA

## Abstract

MicroRNA (miRNA) is a type of endogenous short−stranded ncRNA that influences many biological processes such as animal growth, development and metabolism. The thyroid gland is an important endocrine gland in sheep, and an increasing number of studies have shown that the thyroid gland plays an important role in animal reproduction, but the molecular mechanisms of the thyroid gland in sheep reproduction are poorly understood. In this study, RNA-seq was used to detect transcriptome expression patterns in the thyroid gland between the follicular phase (FP) and luteal phase (LP) in *FecB BB* (MM) and *FecB* ++ (ww) small-tail Han (STH) sheep, respectively, and to identify differentially expressed miRNAs (DEMs) associated with reproduction. Bioinformatic analysis of the target genes of these DEMs revealed that they can be enriched in multiple GO terms associated with the reproductive process in animals and in the KEGG signaling pathway. The miRNA–mRNA coexpression network revealed that oar-miR-133 and oar-miR-370-3p may play an important role in sheep reproduction. The results of the dual-luciferase reporter assay suggest a possible targeting relationship between novel-51 and *TARBP2*. These results provided a novel resource for elucidating regulatory mechanisms underlying STH sheep prolificacy.

## 1. Introduction

STH sheep have excellent characteristics such as fast growth and development, high fertility, perennial estrus, strong disease resistance and genetic stability; reproductive performance is one of the most important economic traits of STH sheep, influenced by factors such as breed, age, nutrition, ovarian cycle and genetics [1]. Genetic factors are intrinsic and controlled by several genes [2]. The *FecB* gene is the most important gene affecting lambing traits in sheep, and it is also the most studied and widely studied gene; our team found that the average lambing number of the *FecB BB* genotype is significantly higher than that of the *FecB* ++ genotype [3].

The thyroid gland is one of the “three glands” in sheep and is involved in most physiological processes of the body. Thyroid follicular epithelial cells are responsible for the synthesis and secretion of thyroid hormones, including triiodothyronine (T3) and thyroxine (T4) [4], which are tyrosine-based compounds containing iodine, and primarily act on nuclear receptors [5]. The thyroid gland primarily relies on T3 and T4 to promote the body’s metabolism and sustain normal growth and development [6]. Thyroid peroxidase (TPO) is a key enzyme in thyroid hormone synthesis, and its gene is specifically expressed in thyroid tissue as a glycosylated heme protein [7]. Disorders of thyroid secretion affect the basal metabolic rate of the organism, which in turn affects the health of the animal [8]. A growing number of studies have demonstrated that thyroid hormones play an irreplaceable role in animal reproduction. T3 and T4 can act directly on tissues such as ovary, placenta and uterus through specific nuclear receptors [9]. In addition, T3 and T4 can also influence the release of gonadotropin-releasing hormone (GnRH) neurons in the hypothalamus through the action of kisspeptins (KISSs) and RFamide-related peptides (RFRPs) to regulate the secretion of LH and FSH to modulate the estrous cycle. GnRH, in turn, regulates the secretion of LH and FSH to regulate the animal’s estrous cycle. In seasonal estrous animals such as sheep, an additional pathway of T3 and T4 action involves the regulation of GnRH secretion by controlling the plasticity of the hypothalamic ventricular membrane cells [10], and through the hypothalamic–pituitary–gonadal axis to impact the follicular and luteal phases in mammals [11], consequently affecting the reproductive process in sheep.

miRNA is a category of noncoding RNA of about 19~22 nucleotides in length, which has a complex occurrence. First, primary miRNA is transcribed in the nucleus, then processed by Drosha enzymes into precursor miRNA with hairpin structure, and finally mature miRNA is formed [12]. The mechanism of action of miRNA in most mammals is base complementary pairing with the 3’UTR of mRNA, thereby inhibiting the translation process of mRNA [13]. A growing number of studies suggest that miRNAs play an important role in animal reproduction. miR-484 regulates granulosa cell function through YAP1-mediated mitochondrial function and apoptosis and reduces human ovarian reserves [14]. Transfection of miR-155 into the oocyte–corona cumulus complex (COC) of *B6D2F1* female mice for in vitro culture revealed that miR-155 inhibited oocyte expansion and oocyte maturation [15]. In addition, miRNAs play a crucial role in the regulation of thyroid hormone secretion, studies have demonstrated that rno-miR-224-5p can modulate thyroid hormone synthesis in mice by directly or indirectly targeting type 1 deiodinase (DIO1) and type 3 deiodinase (DIO3) [16]. Zhang et al. [17] found several differentially expressed miRNAs in the thyroid gland of rats with chronic hypothyroidism and high iodine-induced hypothyroidism. These miRNAs could regulate the expression of key genes affecting the synthesis of thyroid hormones, such as *NIS*, pendrin, *TPO*, *MCT8*, *TSHR*, *TSH*α and *TSH*β, which suggests that miRNAs play a very important role in the metabolism of the thyroid gland. In the context of sheep reproduction, Liu et al. [18] detected a total of 2968 DEGs and 99 DEMs in granulosa cells of high- and low-fertility goats using the RNA-seq technique, and these DEGs and DEMs were found to be significantly enriched in signaling pathways involved in animal reproductive processes, including the PI3K-Akt signaling pathway and JAK-STAT, etc. Through further validation, it was discovered that chi-miR-493-3p regulates ovarian development and growth in goats by modulating the expression of the *JAK3* gene. Di et al. [19] identified 427 miRNAs in the pineal gland of sheep with different estrous cycles and revealed that miR-89 can regulate the reproductive process in sheep by targeting the *AANAT* gene. An et al. [20] conducted an RNA-seq analysis of ovarian tissues from single- and multi-lamb dairy goats, identified several miRNAs involved in the reproductive process, and subsequent study found that miR-101-3p targets the *STC1* gene to regulate the growth process of goat ovaries.

In this study, we identified differentially expressed miRNAs and predicted their potential functions related to reproduction using transcriptomic analysis of the thyroid gland of STH sheep with follicular- phase and luteal-phase *FecB BB* and *FecB* ++ genotypes. It provides a new resource to better understand the molecular mechanisms of miRNA regulation of reproduction in sheep.

## 2. Materials and Methods

### 2.1. Animals and Sample Collection

The *FecB* genotype was identified using TaqMan probes from male jugular vein blood collection from 890 healthy infertile sheep aged 2–4 years from a core breeding herd in the Luxi region of Shandong, China. Twelve STH sheep were selected for the experiment (six *FecB BB* genotypes and six *FecB ++* genotypes). All test ewes were treated for simultaneous estrus and CIDR plugs were placed in the vagina for 12 d and then removed. Fifty hours after bolus withdrawal, three *FecB BB* genotype (MM) and three *FecB ++* genotype (ww) ewes were euthanized, and thyroid tissue was collected. Seven days after bolus withdrawal, three additional MM-type and three ww-type ewes were euthanized, and thyroid tissue was collected. The collected samples were temporarily stored in liquid nitrogen. They were returned to the laboratory and immediately stored in a −80 °C refrigerator for later experiments. The 12 thyroid tissues were divided into 4 groups: follicular-phase *FecB BB* genotype sheep (MM−FT), luteal-phase *FecB BB* genotype sheep (MM−LT), follicular-phase *FecB ++* genotype sheep (ww−FT) and follicular-phase *FecB ++* genotype sheep (ww−LT) for transcriptome sequencing.

### 2.2. RNA Extraction, Library Construction and Sequencing

According to the requirements of the sequencing company, 3 μg of total RNA that passed the test was selected as input material for miRNA library construction, and then miRNA libraries were constructed according to the operating instructions of the Small RNA Sample Pre Kit (NEB, Ipswich, MA, USA). The specific steps were as follows: taking advantage of the special structure of the 3’ end and 5’ end of small RNA (complete phosphate group at the 5’ end and hydroxyl group at the 3’ end), we used total RNA as a starting sample, directly added the splice to both ends of small RNA, and then synthesized cDNA using reverse transcription. The target DNA fragments were subsequently amplified using PCR, separated using PAGE gel electrophoresis, and recovered using gel cutting as a cDNA library. The quality and quantity of cDNA libraries were characterized using Qubit 2.0 and Agilent 2100 to finally obtain miRNA libraries. Afterward, the library was sequenced on the Illumina Hiseq2500 platform to generate 50 bp single-end (SE50) reads.

### 2.3. Sequencing Data Filtering and Comparative Analysis

The raw reads obtained from sequencing were removed using fine filtering methods to remove interfering information. The steps of data filtering are as follows: (1) Remove low-quality reads by filtering out the sequencing read when the number of low-quality (≤20) bases contained in the sequencing read exceeds 50% of the length ratio of those read. (2) Remove the reads containing a high proportion of N. Filter out the sequencing reads when they contain more than 10% of the proportion of the length of the read. (3) Remove reads with 5’ joint contamination. (4) Remove reads without 3’ splice sequences and insertion fragments. (5) Remove the reads containing consecutive A/T/G/C. (6) Remove the reads with abnormal final length.

After filtering the data using the above steps, we obtained a large number of small RNA (sRNA) sequences. Clean reads of length 18–35 nt were used for the subsequent analysis. The sRNAs were compared with the reference genome and the sRNAs and their distributions in the genome were compared for each sample. Bowtie v1.0.1 was used to identify known miRNAs by comparing the clean reads of each sample to the miRNA sequences in the miRBase v22 database. The clean reads of miRNA sequences that were not matched to the miRbase database were matched to data from Rfam v14.2 or Repeat Masker database for sRNA taxonomic annotation. Based on the signature hairpin structure of miRNA precursor molecules, novel miRNAs in the samples were predicted based on mirEvo/miRDeep V2.0.0.5 analysis by probing the secondary structure, Dicer enzyme cleavage site information and minimum free energy of unannotated small RNAs in the previous step.

### 2.4. Differential Expression Analysis

The DESeq2 R package was used to analyze differential miRNAs for different comparison combinations based on TPM values. Quantities were pooled and DEMs were screened at both foldchange and *p*-value levels of assessment. *p* < 0.05 and |log2(foldchange)| > 1 were set as the threshold for significant DEMs.

### 2.5. Target Gene Prediction of miRNAs

The target genes of miRNAs were predicted using miRanda (3.3a), RNAhyribd (https://bibiserv.cebitec.uni-bielefeld.de/rnahybrid/., accessed on 12 February 2023) and PITA (https://genie.weizmann.ac.il/pubs/mir07/mir07_dyn_data.html., accessed on 20 February 2023) online website, and the intersection of the three databases prediction results was taken as the target genes of DEMs in this study (the number of intersecting genes predicted using the three databases types in the MM−LT vs. ww−LT group was 0, so no analysis was performed in this paper); GO and KEGG analysis of target genes of differentially expressed miRNAs were performed using the DAVID online website (https://david.ncifcrf.gov., accessed on 10 Match 2023) and were considered significantly enriched if *p* < 0.05 in the pathway.

### 2.6. Construction of miRNA-mRNA Co-Expression Network

The miRNA-mRNA co-expression network was visualized using Cytoscape (V3.9.0) software.

### 2.7. Validation of Sequencing Data

#### 2.7.1. Reverse Transcription of miRNA

After extracting the total RNA of the samples, the reverse transcription operation of miRNA was performed using the reverse transcription kit of Tiangen (Beijing, China), and the reverse transcription system is shown in Table 1.

The reaction conditions were as follows: 42 °C, 60 min; 95 °C, 3 min. The cDNA that were obtained after the reverse transcription were diluted 5 times and then stored at −20 °C.

#### 2.7.2. Primer Design and Synthesis

Reverse primers are provided in the kit, and based on the sequences of mature miRNAs, we used Primer Premier 5.0 software to design forward primers for miRNAs (Table 2) for subsequent qPCR, with U6 as the internal reference.

#### 2.7.3. RT-qPCR Analysis of miRNA

Fluorescent quantitative PCR was performed using Tiangen’s Fluorescent Quantitative Assay Kit (TIANGEN, Beijing, China). The fluorescence quantitative PCR system is shown in Table 3.

The reaction was performed by placing the configured mixture in a Roche Light Cycler^®^ 480II (Roche Applied Science, Mannheim, Germany) fluorescent quantitative PCR instrument according to the fluorescent quantitative PCR system, and the reaction conditions are shown in Table 4. 

### 2.8. Carrier Construction

The *TARBP2* dual luciferase vector was constructed by selecting the pmirGLO vector and designing PCR primers according to the sequence matching novel-51 in the NCBI database of the *TARBP2−*3’UTR sequence, adding the SacⅠenzyme cut site GAGCTC upstream and the Xho I enzyme cut site CTCGAG downstream of the PCR primers. The remaining steps were performed as above, and finally the *TARBP2−*3’UTR wild-type vector (pmirGLO−*TARBP2−*WT) and *TARBP2−*3’UTR mutant vector (pmirGLO−*TARBP2−*MT) were successfully constructed.

### 2.9. Validation of miRNA–mRNA Targeting Relationship

HEK293T cell lines were inoculated into 12-well cell culture plates containing complete medium (DMEM + 10% FBS + 1.5% double antibody) and incubated in a 37 °C cell culture incubator. Transfection was performed when the cell density reached 70–80%, and the experiment was divided into 4 groups, with 3 replicates set in each group. Group I: pmirGLO−*TARBP2−*WT + novel-51 mimics. Group II: pmirGLO−*TARBP2−*WT + novel-51 mimics NC. Group III: pmirGLO−*TARBP2−*MT + novel-51 mimics. Group IV: pmirGLO−*TARBP2−*MT + novel-51 mimics NC. After transfection, the cells were placed in a cell culture incubator and starved with OPTI-MEM for 6 h. After replacing the complete medium, the culture was continued for 48 h. The supernatant was aspirated and 100 μL of trypsin was added to each well, and the digestion was terminated by adding the appropriate amount of serum after 3 min, and the cells were collected after discarding the supernatant. The double-luciferase activity assay was performed with the TransDetect Double-Luciferase Reporter Assay Kit (Promega, Beijing, China). Seventy-five microliters of Luciferase Reaction Reagent I was added to the cell precipitate according to the instructions. After thorough mixing, the firefly luciferase activity was detected using a Tecan Infiniate 200 Pro multifunctional enzyme marker, and then 75 μL of Luciferase Reaction Reagent II was added after the assay was completed, and the sea kidney luciferase activity was detected after waiting for 20–30 min.

### 2.10. Statistical Analysis

The relative expression of miRNAs was analyzed using an independent samples *t*-test. The dual-luciferase reporter gene data were calculated from the relative luciferase activity data using a one-way ANOVA, and the data complied with the assumption of normality and the assumption of chi-square of variance of the “ANOVA” test. Relative luciferase activity = activity of sea kidney luciferase reporter gene/activity of firefly luciferase reporter gene. *p* < 0.05 indicates a significant difference. Statistical analysis and plots were performed using GraphPad Prism 8.3.0.

## 3. Results

### 3.1. Differential Expression and Analysis of miRNA

**Figure 1:**
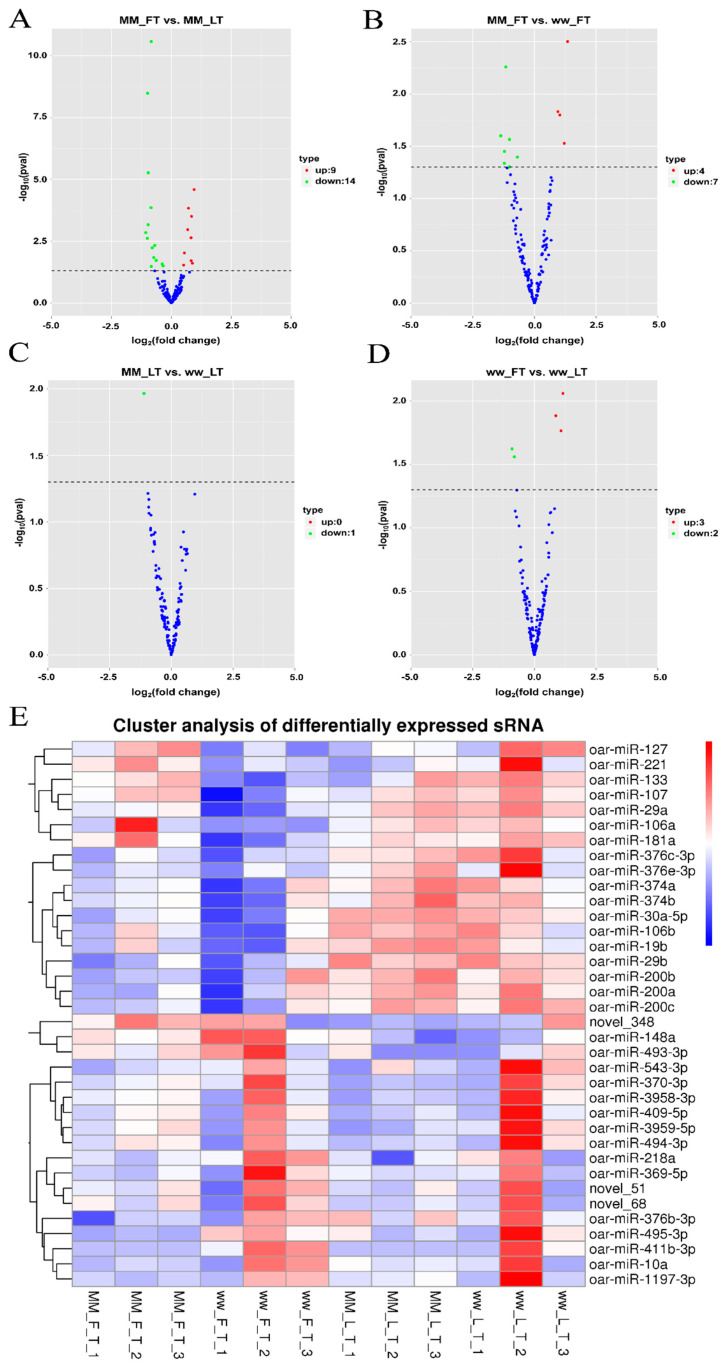
DEMs analysis among four groups. Volcano plots show the up− and downregulation distribution of DEMs in (**A**) MM−FT vs. MM−LT, (**B**) MM−FT vs. ww−FT, (**C**) MM−LT vs. ww−LT and (**D**) ww−FT vs. ww−LT, where red and green represent up− or downregulation, respectively. Heatmap (**E**) shows the expression patterns of the four groups of DEMs.

The MM−FT vs. MM−LT group identified 23 DEMs (9 upregulated and 14 downregulated, Figure 1A). The MM−FT vs. ww−FT group identified 11 DEMs (4 upregulated, 7 downregulated, Figure 1B). One DEM was found in the MM−LT vs. ww−LT group (downregulated, Figure 1C). Five DEMs were found in the ww−LT vs. ww−FT group (two upregulated, three downregulated, Figure 1D). The heat map shows the number and expression pattern of DEMs, and all DEMs were statistically significant (Figure 1E, *p* < 0.05; Supplementary Table S1).

### 3.2. Functional Enrichment Analysis of miRNA Target Genes

**Figure 2:**
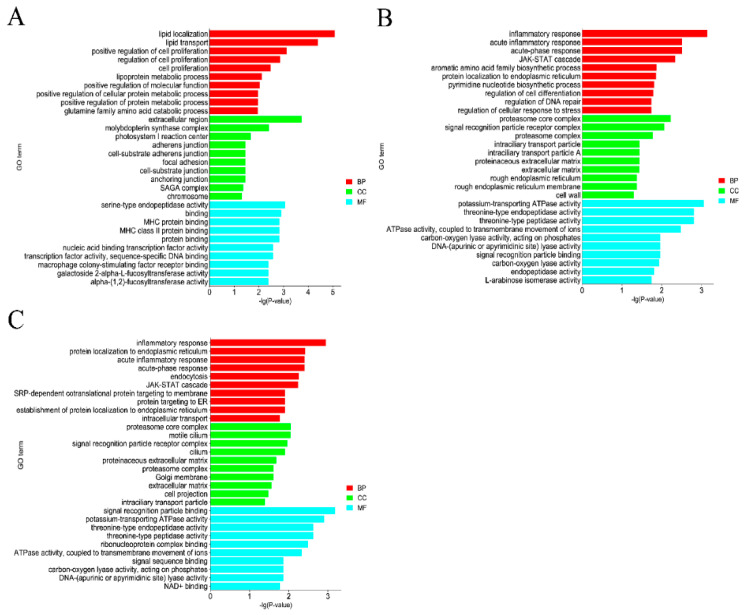
Top 10 enriched GO terms of genes targeted by DELs in four groups. The top 10 enriched GO terms of genes targeted by DELs in (**A**) MM−FT vs. MM−LT (**B**) MM−LT, MM−FT vs. ww−FT, (**C**) ww−FT vs. ww−LT. The horizontal and vertical coordinates represent the GO terms and −lg (*p*−Value) of the enriched genes, respectively.

**Figure 3:**
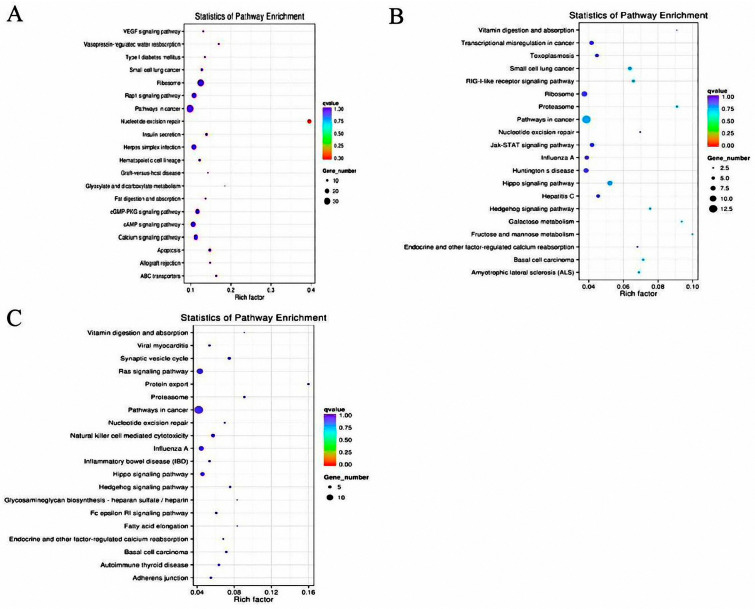
Twenty enriched KEGGs of genes targeted by DEMs in four groups. Twenty enriched KEGG of genes targeted by DEMs in (**A**) MM−FT vs. MM−LT, (**B**) MM−FT vs. ww−FT, (**C**) ww−FT vs. ww−LT. Horizontal and vertical coordinates represent the −lg (*p*−Value) of the enriched genes and KEGG pathway, respectively.

In the GO and KEGG bioinformatics analysis of the identified DEMs, GO enrichment revealed that in the MM−FT vs. MM−LT group (Figure 2A), the most significant GO entry enriched during BP was Lipid localization, with a total of 22 DEMs target genes being enriched; the CC category Extracellular region was the most enriched; the Serine-type endopeptidase activity was the most enriched term in the MF process. The most significant GO entries enriched during BP in both the MM−FT vs. ww−FT group (Figure 2B) and the ww−FT vs. ww−LT group (Figure 2C) were inflammatory responses; the CC category proteasome core complex is the most enriched term in the MM−FT vs. ww−FT group and the ww−FT vs. ww−LT group. The most enriched term in the MM−FT vs. ww−FT group during MF was Potassium-transporting ATPase activity, and the most enriched term in ww−FT vs. ww−LT was Signal recognition particle binding (Supplementary Table S2). KEGG enrichment analysis revealed that the most significantly enriched signaling pathways in the three groups of DEMs target genes were Nucleotide excision repair, Proteasome and Protein export signaling pathway, JAK-STAT signaling pathway and Hippo signaling pathway (Figure 3, and Supplementary Table S3).

### 3.3. Analysis of miRNA–mRNA Co-Expression Network

**Figure 4:**
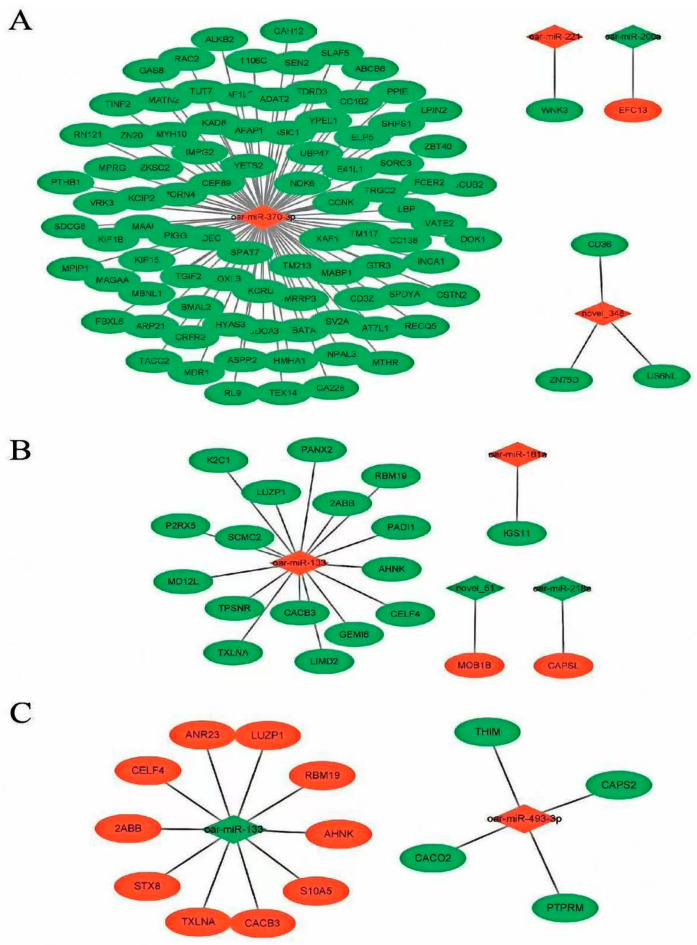
DEM−DEG network interaction analysis. The DEM–DEG network between (**A**) MM−FT vs. MM−LT, (**B**) MM−FT vs. ww−FT, (**C**) ww−FT vs. ww−LT. Note: Nodes represent DEMs or DEGs, and edges represent the interaction between DEMs and DEGs. Red represents upregulation and green represents downregulation. Circular and inverted rhombus represents DEGs and DEMs, respectively.

To better understand the role of DEGs in the thyroid gland of STH sheep, we constructed an miRNA–mRNA co-expression network (Figure 4 and Supplementary Table S4). A total of 123 DEGs were targeted by 10 DEMs in the co-expression network. Among them, oar-miR-370-3p and oar-miR-133 targeted the most regulated mRNAs, implying that oar-miR-370-3p and oar-miR-133 play an important role in the reproductive process of STH sheep.

### 3.4. Validation of Sequencing Data

**Figure 5:**
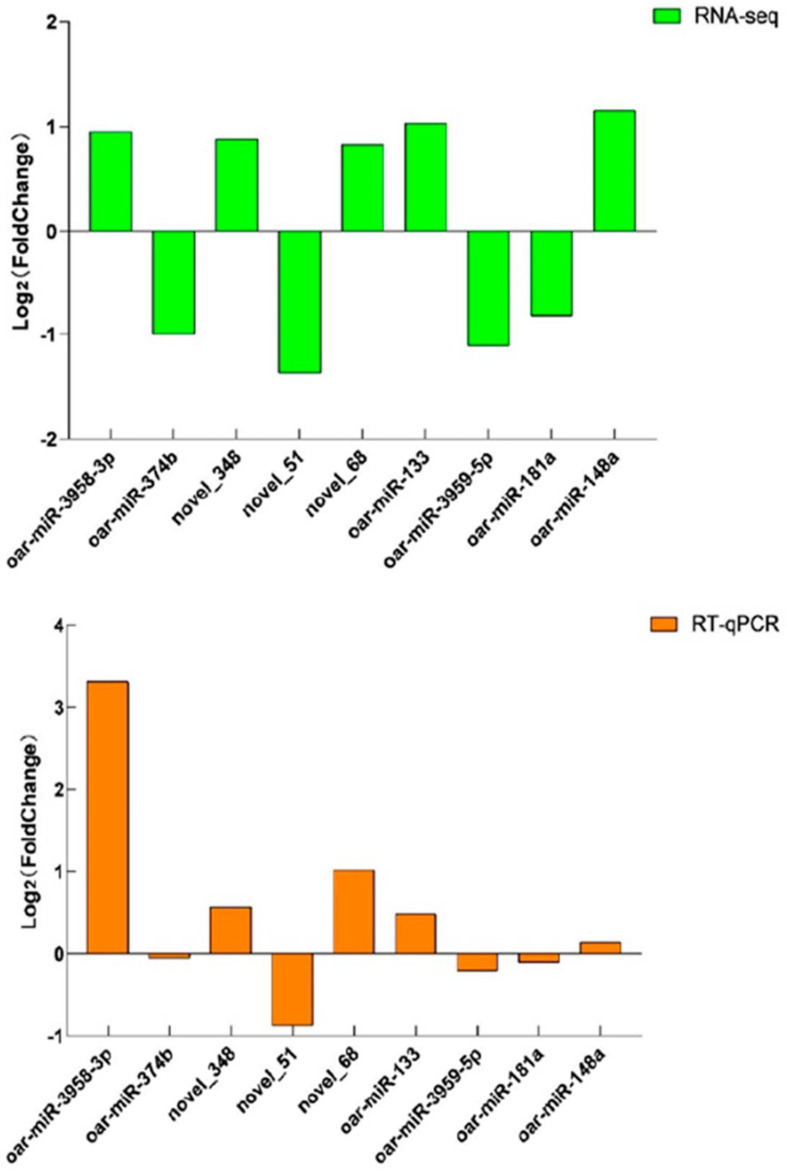
RT−qPCR verification of DEMs. RT−qPCR verified the expression trend of DEMs in MM−FT vs. MM−LT, MM−FT vs. ww−FT, MM−LT vs. ww−LT, and ww−FT vs. ww−LT.

To verify the accuracy of the sequencing data, we randomly selected nine miRNAs: oar-miR-3958-3p, oar-miR-374b, novel_348, novel_51, novel_68, oar-miR-133, oar-miR-3959-5p, oar-miR-181a and oar-miR-148a for RT-qPCR test validation, and the results showed that the RT-qPCR trends and RNA-seq expression trends were consistent, indicating that the sequencing results were reliable (Figure 5 and Supplementary Table S5).

### 3.5. Plasmid Construction and Dual-Luciferase Experimental Validation

**Figure 6:**
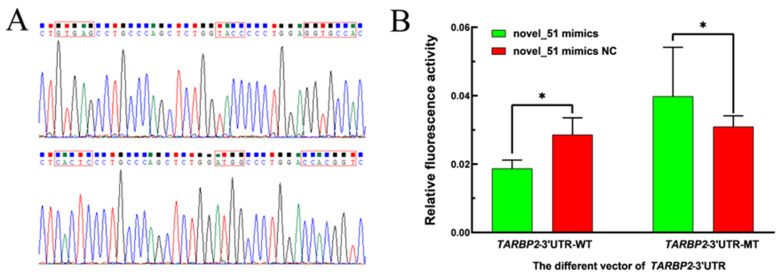
Plasmid construction and double-luciferase activity detection results. (**A**) Sequencing results of *TARBP2*−3’UTR−WT and MT plasmids. (**B**) Relative fluorescence activity was detected after cotransfection of novel-51 mimics, novel-51 mimics NC, *TARBP2*−WT and *TARBP2*−MT into 293T cells. Note: “*” denotes significant differences in expression (*p* < 0.05).

Sequencing of recombinant plasmid DNA showed that the sequences of overexpression plasmid and wild-type plasmid were consistent with the reference sequence, and the mutant plasmid sequences were GTGAG mutated to CACTC, TACC mutated to ATGG, and GGTGCCA mutated to CCACGGT for *TARBP2−*3’UTR (Figure 6A); The results of the dual-luciferase assay showed that the relative fluorescence activities of pmirGLO−*TARBP2−*WT + novel-51 mimics group (Figure 6B) (*p* < 0.05 and Supplementary Tables S6 and S7).

## 4. Discussion

In this study, DEMs were screened for the first time in thyroid tissues from follicular- and luteal-phase STH sheep of *FecB BB* and *FecB* ++ genotype. Bioinformatic analysis of DEMs target genes revealed that miR-133, miR-370-3p, miR-29, miR-181a and novel-51 target genes were significantly enriched in signaling pathways related to the reproductive process. 

miR-133b has been reported to impact estrogen synthesis by targeting *FOXL2* in mice [21], and may also be involved in early oogenesis in tilapia through regulation of *TAGLN2* expression [22]. miR-2 and miR-133 bind to the 3’UTR of the cyclin B gene of the crab Eriocheir sinensis to regulate its oocyte meiosis [23]. Liu et al. [24] identified circRNAs in ovarian tissues of goats with different reproductive cycles using the RNA-seq technique. They discovered that the differentially identified circRNAs could be targeted by miR-133, indicating that miR-133 plays an important role in the reproductive process of goats. Moreover, miR-370-3p has been recognized as an important regulator of gene expression in animal reproduction, specifically acting as a negative regulator of endometriotic cell proliferation [25]. Using microarray technology to determine miRNA expression patterns in the endometrium of ewes at different periparturient stages, we identified significant differences in miR-370-3p expression. These findings suggest that miR-370-3p may function as a regulator of early pregnancy establishment and maintenance in sheep [26]. RNA-seq analysis of the adrenal glands of STH sheep [27] with different *FecB* genotypes and thyroid tissues of Sunite sheep [28] with different photoperiods revealed miR-370-3p as the central element of the network. These findings suggest that miR-370-3p could potentially impact the reproductive process in sheep. 

In addition, miR-29 and miR-181a have also been reported to be involved in the reproductive process of animals. In the context of lncRNA Xist, miR-29 obstructs the maturation of miR-23b-3p/miR-29a-3p by inhibiting pre-miR-23b/pre-miR-29a to the cytoplasm and attenuates miR-23b-3p/miR-29a-3p inhibition of *STX17*, leading to oocyte autophagy in the periparturient ovary, resulting in massive oocyte loss [29]. Knockout of miR-29a/b1 gene in mice leads to abnormal expression of numerous proteins involved in vesicular transit and cytokinesis in the mouse pituitary gland, leading to impaired ovulation and compromised LH secretion [30]. miR-29b regulates DNA methylation during early porcine embryonic development by targeting DNMT3A/3B and TET1/2/3 [31]. Additionally, research has revealed that miR-29 might regulate early bovine embryogenesis by regulating the expression of *NPM2* [32]. miR-181a has been identified to promotes follicle development and ovulation in mice by targeting the *AMH* gene, thus preventing follicular atresia [33]. Zayed et al. [34] used RNA-seq to identify miRNA expression profiles in zebrafish stage IIIa follicle cells and stage IIIb follicle cells and found differences in miR-181a expression, implying that miR-181a is an important regulator of follicle development and oocyte maturation in zebrafish. 

GO enrichment analysis revealed that DEMs target genes could be enriched in extracellular regions, serine-type endopeptidase activity, inflammatory response, proteasome core complex, potassium-transporting ATPase activity, and signal recognition particle binding terms. KEGG enrichment analysis revealed that these DEMs put genes significantly enriched in signaling pathways involved in the reproductive process such as the Hippo signaling pathway, cGMP-PKG signaling pathway, VEGF signaling pathway and JAK-STAT signaling pathway. The Hippo signaling pathway plays vital role in embryonic development, organ size regulation and carcinogenesis. Yes-associated protein 1 (YAP1), as a significant constituent of the Hippo signaling pathway, holds particular importance. The Hippo pathway is crucial in regulating proliferation of bovine ovarian granulosa cells and the synthesis of estradiol, thereby contributing to the maintenance of normal follicular development [35]. Inhibition of the Hippo signaling pathway inhibits GnRH-induced ovulation in cattle, indicating that this pathway indeed plays a crucial role in animal ovulation [36]. Additionally, the Hippo signaling pathway might regulate the secretion of luteinizing hormone in the mouse pituitary gland, consequently regulating the reproductive process in mice [37].

The cGMP-PKG signaling pathway holds paramount important in animals. Li et al. [38] used RNA-seq to identify ovarian tissues from sheep with the *FecB BB* genotype and *FecB* ++ genotype and identified several differential miRNAs, which were found to be significantly enriched in the cGMP-PKG signaling pathway for their target genes. Activation of cGMP-PKG signaling in spermatozoa increases Ca2+ and tyrosine-phosphorylated proteins, which in turn promotes hyperactivation and induces acrosome responses ultimately facilitating sperm capacitation [39]. Studies have shown that nitric oxide activates the cGMP-PKG pathway by upregulating glucose transporter-1 (*GLUT1*) and glucose transporter-4 (*GLUT4*) in granulosa cells, which increases cellular glucose uptake and promotes granulosa cell development [40].

Vascular endothelial growth factor (VEGF) is thought to exert beneficial effects on sheep oocyte maturation in vitro and subsequent early embryonic development by binding to its main receptor, KDR. This binding event activates the MAPK signaling pathway, thus promoting the maturation of sheep oocytes [41]. The observation revealed that heat stress could elevate miR-33 expression in tilapia, diminish follicle-stimulating hormone and 17β-estradiol levels via the miR-33-TGFβ1I1 axis, and increase apoptosis by inhibition VEGF signaling, ultimately leading to follicular atresia [42]. Furthermore, studies have indicated the FSH-HIF-1α-VEGF pathway is responsible for ovulation and oocyte health in mice, while it exerts minimal influence on follicle growth [43].

The JAK-STAT signaling pathway sustains and stimulates the growth of primordial follicles in women, thereby regulating the oocyte growth process and consequently female fertility [44,45]. Additionally, leptin inhibits uterine contractions in mice by stimulating the JAK-STAT signaling pathway [46]. The characterization of miRNA and mRNA expression profiles in hypothalamic tissues of high- and low-follicular fertility goats reveals that the identified differential expressed genes are significantly enriched in the JAK-STAT signaling pathway [47,48].

To gain a deeper understanding of the mechanisms governed by miRNAs in the reproductive process of STH sheep, we constructed a miRNA-mRNA co-expression network. Within this network, we discovered that oar-miR-133 and oar-miR-370-3p targeted the largest number of genes, implying that oar-miR-133 and oar-miR-370-3p play important roles in the thyroid gland of STH sheep. In addition, we confirmed the targeting relationship between novel-51 and its target gene *TARBP2*, as well as between oar-miR-370-3p and its target gene *COL4A3* [49], where the *COL4A3* gene encodes type IV collagen α 3 protein [50]. The differential gene expression profiles of the endometrium in high- and low-fertility heifers were identified using microarray technology, which highlighted *COL4A3* as the significantly differentially expressed gene, implying that the *COL4A3* gene may affect the late development process of the corpus luteum and fertility in heifers [51]. In this study, COL4A3 was also enriched in signaling pathways such as the relaxin signaling pathway and *PI3K*-Akt, suggesting that oar-miR-370-3p may affect PI3K-Akt signaling pathway by targeting *COL4A3*, which in turn regulates thyroid tissue. RNA-seq analysis of the endometrium of sows at 12, 16 and 20 days of gestation revealed *TARBP2* as the gene with significant differences in expression, indicating that *TARBP2* plays an important role in the reproduction of sows [52]. In the present study, a possible targeting relationship between novel-51 and TARBP2, oar-miR-370-3p and *COL4A3* was tentatively identified using a dual-luciferase reporter assay, providing a basis for further understanding of the role played by the thyroid gland in sheep reproduction.

## 5. Conclusions

In this study, multiple DEMs were found in the thyroid glands of STH sheep with follicular- and luteal-phase *FecB BB* genotypes and *FecB* ++ genotypes. These DEM genes, which can be significantly enriched in multiple signaling pathways, are involved in the reproductive process of animals. miRNA−mRNA co-expression network analysis revealed that oar-miR-133 and oar-miR-370-3p play important roles in sheep reproduction. Dual-luciferase assays suggest a possible targeting relationship between novel-51 and *TARBP2*, oar-miR-370 and *COL4A3*. Further functional validation of its function in the sheep thyroid is necessary.

## Figures and Tables

**Table 1:** The amount of miRNA reverse transcription reagents used.

Reagents	Volume
Total RNA (500 ng/µL)	2 µL
2×miRNA RT Reaction Buffer	10 µL
miRNA RT Enzyme Mix	2 µL
RNase-Free ddH_2_O	6 µL

**Table 2:** The primer sequences designed for real-time fluorescence quantification.

Gene Name	Primer Sequences (5′-3′)	Tm (°C)
*oar-miR-3958-3p*	F: CGCAGATATTGCACGGTTGATCTCT	60
*oar-miR-374b*	F: CGCCGCATATAATACAACCTGC	60
*novel_348*	F: TCTGGTGCTTAGACTCTGTGCT	60
*novel_51*	F: GCTATGGCACTGGTAGAATTCACT	60
*novel_68*	F: GTTTGGCACTAGCACATTTTTGCT	60
*oar-miR-133*	F: TTGGTCCCCTTCAACCAGCTGT	60
*oar-miR-3959-5p*	F: CGCGGTTGATCAGAGAACATAC	60
*oar-miR-181a*	F: AACATTCAACGCTGTCGGTGAGT	60
*oar-miR-148a*	F: GCTCAGTGCACTACAGAACTTTGT	60
*U6*	F: CCAAGGATGACACGCAAATTCG	60

**Table 3:** qPCR reagent usage.

Reagents	Volume
2×miRcute plus miRNA premix	10 µL
Forward primer	0.4 µL
Reverse primer (provided in the reagent kit)	0.4 µL
miRNA first strand cDNA	2 µL
RNase-free ddH_2_O	7.2 µL

**Table 4:** Condition setting of qPCR.

Cycles	Temperature (°C)	Time	Reaction Content
1×	95	15 min	Starting template denaturation
5×	94	20 s	Enrichment of target miRNAs
	64	30 s	
	72	34 s	
40×	94	20 s	Template denaturation in PCR cycles
	60	34 s	Annealing, extension

## Data Availability

All data are included in this paper.

## Supplementary Materials

The following supporting information can be downloaded at: https://www.mdpi.com/article/10.3390/genes14112024/s1, Table S1: Information of total miRNAs in four groups. Table S2: Total set of DEMs was up- and downregulated in four groups. Table S3: GO enrichment of differentially expressed DEMs targets in four groups. Table S4: KEGG enrichment pathways for differentially expressed DEMs targets in four groups. Table S5: Co-expression details of miRNA–mRNA network in four groups. Table S6: qPCR data of DEMs in four groups. Table S7: Dual-luciferase report experimental data.
