# Supplementary material for: Thyroid Transcriptomics Revealed the Reproductive Regulation of miRNA in the Follicular and Luteal Phases in Small-Tail Han Sheep with Different FecB Genotypes
Source: Genes (Basel). 2023 Oct 30;14(11):2024. doi: 10.3390/genes14112024 (PMC10671830; doi:10.3390/genes14112024)
Supplement: Supplementary file 1 [file genes-14-02024-s001.zip › Description of supplementary materials.pdf]

Supplementary Table S1: Total set of DEMs was up-and-down-regulated in four groups.

Supplementary Table S2: GO enrichment of differentially expressed DEMs targets in four groups.

Supplementary Table S3: KEGG enrichment pathways for differentially expressed DEMs targets in four groups.

Supplementary Table S4: Co-expression details of DEMs-DEGs after DEMs targets coincided with DEGs in four groups.

Supplementary Table S5: QPCR data of DEMs in four groups.

Supplementary Table S6: Sequence of the constructed vector.

Supplementary Table S7: Dual luciferase report experimental data.
